# Association of Exposure to Police Violence With Prevalence of Mental Health Symptoms Among Urban Residents in the United States

**DOI:** 10.1001/jamanetworkopen.2018.4945

**Published:** 2018-11-21

**Authors:** Jordan E. DeVylder, Hyun-Jin Jun, Lisa Fedina, Daniel Coleman, Deidre Anglin, Courtney Cogburn, Bruce Link, Richard P. Barth

**Affiliations:** 1 Graduate School of Social Service, Fordham University, New York, New York; 2 School of Social Work, University of Maryland, Baltimore; 3 School of Social Work, University of Michigan, Ann Arbor; 4 Graduate Center, City College of New York, New York; 5 School of Social Work, Columbia University, New York, New York; 6 School of Public Policy, University of California Riverside, Riverside

## Abstract

**Importance:**

Police violence is reportedly widespread in the United States and may pose a significant risk to public mental health.

**Objective:**

To examine the association between 12-month exposure to police violence and concurrent mental health symptoms independent of trauma history, crime involvement, and other forms of interpersonal violence exposure.

**Design, Setting, and Participants:**

This cross-sectional, general population survey study of 1221 eligible adults was conducted in Baltimore, Maryland, and New York City, New York, from October through December 2017. Participants were identified through Qualtrics panels, an internet-based survey administration service using quota sampling.

**Exposures:**

Past 12-month exposure to police violence, assessed using the Police Practices Inventory. Subtypes of violence exposure were coded according to the World Health Organization domains of violence (ie, physical, sexual, psychological, and neglectful).

**Main Outcomes and Measures:**

Current Kessler Screening Scale for Psychological Distress (K6) score, past 12-month psychotic experiences (World Health Organization Composite International Diagnostic Interview), and past 12-month suicidal ideation and attempts.

**Results:**

Of 1221 eligible participants, there were 1000 respondents (81.9% participation rate). The sample matched the adult population of included cities on race/ethnicity (non-Hispanic white, 339 [33.9%]; non-Hispanic black/African American, 390 [39.0%]; Hispanic/Latino, 178 [17.8%]; other, 93 [9.3%]), age (mean [SD], 39.8 [15.2] years), and gender (women, 600 [60.0%]; men, 394 [39.4%]; transgender, 6 [0.6%]) within 10% above or beyond 2010 census distributions. Twelve-month prevalence of police violence was 3.2% for sexual violence, 7.5% for physical violence without a weapon, 4.6% for physical violence with a weapon, 13.2% for psychological violence, and 14.9% for neglect. Police violence exposures were higher among men, people of color, and those identified as homosexual or transgender. Respondents reported suicidal ideation (9.1%), suicide attempts (3.1%), and psychotic experiences (20.6%). The mean (SD) K6 score was 5.8 (6.1). All mental health outcomes were associated with police violence exposure in adjusted logistic regression analyses. Physical violence with a weapon and sexual violence were associated with greater odds of psychotic experiences (odds ratio [95% CI]: 4.34 [2.05-9.18] for physical violence with a weapon; 6.61 [2.52-17.36] for sexual violence), suicide attempts (odds ratio [95% CI]: 7.30 [2.94-18.14] for physical violence with a weapon; 6.63 [2.64-16.64] for sexual violence), and suicidal ideation (odds ratio [95% CI]: 2.72 [1.30-5.68] for physical violence with a weapon; 3.76 [1.72-8.20] for sexual violence).

**Conclusions and Relevance:**

Police violence was commonly reported, especially among racial/ethnic and sexual minorities. Associations between violence and mental health outcomes did not appear to be explained by confounding factors and appeared to be especially pronounced for assaultive forms of violence.

## Key Points

**Question:**

What is the prevalence of police violence exposure in cities in the United States, and what is the association of police violence exposure with adverse mental health outcomes independent of other forms of trauma exposure and crime involvement?

**Findings:**

In this cross-sectional, general population survey study of 1000 adults residing in Baltimore, Maryland, and New York City, New York, police violence exposure was reported at a prevalence ranging from 3% for sexual violence to 15% for neglect, inequitably distributed across demographic groups, and was associated with concurrent mental health symptoms.

**Meaning:**

The findings suggest that police violence is experienced by many urban residents in the United States and may be associated with mental health disparities.

## Teaser

This general population survey study evaluates the association between past 12-month exposure to police violence and concurrent mental health symptoms among urban residents in 2 US cities.

## Introduction

Police violence has been increasingly recognized as a public health issue in the United States, particularly after the rapid increase in public awareness through smartphone technology.^1,2,3^ In general, data on police violence have not been systematically or reliably collected by US police departments, federal criminal justice regulatory agencies, or in nationally representative surveys. Although research on fatalities by police^4^ has benefited from crowd-sourced attempts to comprehensively document these incidents,^5^ awareness of nonfatal incidents is dependent on self-reported data from civilians, which has only recently been systematically collected.^6,7,8,9,10^ Among these efforts, few studies have assessed the association of mental health with nonfatal police violence exposures. This assessment is needed to develop comprehensive public health interventions aimed at preventing police violence and its mental health consequences.

In research using self-reported data from civilians, 2 studies have assessed the psychiatric implications of police encounters and found that unfair treatment^11^ and aggressive policing^12^ were significantly associated with the occurrence of mood and anxiety disorders. Furthermore, male residents of neighborhoods in New York City, New York, in which “stop and frisk” policing was common self-reported greater psychological distress than those living in neighborhoods where stop and frisk was rare.^13^ Apart from these studies, to our knowledge, no prior data from general population samples exist on the type and prevalence of multiple dimensions of police violence exposures (physical, sexual, psychological, and neglect) and whether these exposures are associated with multiple mental health outcomes. The initial Survey of Police-Public Encounters (SPPE I) sought to rectify this lack of evidence by validating novel measures of police violence exposure and assessing the lifetime prevalence of police violence and its association with mental health in 4 US cities.^6^ That study found that minor forms of police violence (psychological and neglectful) were common (approximately 20% lifetime prevalence among adults in the general population), more severe forms of violence (sexual, physical with a weapon) were not rare (approximate lifetime prevalence among adults in the general population, 3% for sexual violence and 6% for physical violence with a weapon), and all forms of violence exposure were associated with greater odds of psychological distress and depression,^6^ suicide attempts,^7^ and subclinical psychotic experiences.^8,10^ Furthermore, although there was a higher prevalence of exposure among young adults, males, and black and Latino respondents, all groups experienced some degree of exposure to untoward treatment from the police,^6^ consistent with self-reported data collected by the US Bureau of Justice Statistics.^14^

The SPPE I study provided an initial understanding of the scope of police violence but did not reliably assess recent (eg, 12-month) police violence exposure as opposed to lifetime exposure. Second, police violence exposure is subject to confounding^15^ because it is more common among those who have been involved in crime^6^ or exposed to other forms of violence and trauma.^9^ These constructs were not adequately measured in the SPPE I.

This survey study aimed to present data on the 12-month and lifetime prevalence of police violence exposure and to assess associations between each subtype of police violence exposure and 4 major mental health outcomes (severe distress, suicidal ideation, suicide attempts, and psychotic experiences), while adjusting for likely confounders including crime involvement, intimate partner violence exposure, and adverse childhood experiences.

## Methods

### Procedures and Participants

Study procedures were approved by the institutional review board at the University of Maryland, Baltimore. Potential participants (N = 1221) were provided information regarding the purpose of the study and were given the option to exit the site or to consent to complete the survey online. Participants were financially compensated at rates determined by Qualtrics, an online survey administration service maintaining a database of several million US residents. This survey study followed the American Association for Public Opinion Research (AAPOR) reporting guideline for conducting web-based surveys using nonprobability internet panels and the Strengthening the Reporting of Observational Studies in Epidemiology (STROBE) reporting guideline for reporting cross-sectional results. The second Survey of Police-Public Encounters (SPPE II), a cross-sectional, general population survey, was conducted from October through December 2017. The survey assessed self-reported police violence, mental health symptoms, crime involvement, adverse childhood experiences, and intimate partner violence exposure. Research participants 18 years and older were recruited from Baltimore, Maryland, and New York City, New York, based on geographic household data provided by Qualtrics panels. Epidemiological research has increasingly used online survey panels, especially Qualtrics panels,^16,17,18,19^ to obtain general population samples.

Qualtrics used a quota sampling methods by using a combination of demographic screening questions and recruitment quotas to reflect the demographic makeup of each city (ie, ±10% of 2010 census distributions for age, sex, and race/ethnicity in each city), with a predetermined target sample size of 1000 completed surveys. When limits were met for their particular demographic group within each city’s boundaries (defined by GPS coordinates at time of survey completion and confirmed with self-reported city of residence), no additional participants were asked to complete the survey. Participants who did not complete the survey were excluded from subgroup quotas.

### Measures

#### Mental Health Outcomes

Psychological distress was measured using the Kessler Screening Scale for Psychological Distress (K6) score, a brief assessment of clinically significant psychological difficulties, including hopelessness, worthlessness, nervousness, agitation, and depression, over the previous 4 weeks. The internal consistency of the K6 (Cronbach α = 0.92) was excellent in this sample.

Past 12-month psychotic experiences were assessed using the 4-item self-report version (3.0) of the World Health Organization Composite International Diagnostic Interview (WHO-CIDI) psychosis module, recoded as a binary variable (any psychotic experience: yes or no) for this study.^20,21^ The WHO-CIDI psychosis screen is commonly used in epidemiological studies to measure psychotic experiences.^22,23,24,25^

Past 12-month suicidal ideation (in the past 12 months, have you ever seriously thought about committing suicide?) and suicide attempts (in the past 12 months, have you ever attempted suicide?) were measured each with a single item consisting of 3 response categories (yes, no, or unsure), consistent with the measures used in national adult population surveys such as the National Survey on Drug Use and Health (NSDUH) and the National Comorbidity Survey (NCS).^26^ Response options were dichotomized by combining the unsure and no responses.

#### Police Violence Exposure

Past experiences of police violence were assessed using the 27-item Police Practices Inventory (PPI),^6^ developed based on the 4 WHO domains of violence (ie, physical, sexual, psychological, and neglect).^27^ Of note, the WHO (and therefore, this measure) uses a broad definition of violence that includes nonphysical and sexual forms, particularly when coming from individuals or groups in power, based on the rationale that if someone in power is expected to protect others from violence and neglects to do so, that lack of intervention can itself be considered a violent act. The PPI variables included in the present study consisted of 5 dichotomous items: (1) physical violence with a weapon (has a police officer ever used a gun, baton, taser, or other weapon on you?), (2) physical violence without a weapon (has a police officer ever hit, punched, kicked, dragged, beat, or otherwise used physical force against you?), (3) psychological violence (has a police officer ever engaged in nonphysical aggression toward you, including threatening, intimidating, stopping you without probable cause, or using slurs?), (4) sexual violence (has a police officer ever forced inappropriate sexual contact on you, including while conducting a body search in a public place?), and (5) neglect (have you ever called or summoned the police for assistance and the police either did not respond, responded too late, or responded inappropriately?). Each was coded as a separate exposure variable. Respondents were then asked to report how many times in the past 12 months they experienced each domain of PPI, which were recoded into binary variables (any exposure in past 12 months: yes or no). Lifetime and 12-month prevalence rates are reported, although primary analyses focus on 12-month exposure.

#### Sociodemographics

Survey participants self-reported sociodemographics including gender identity (male, female, transgender, or other), age (18-24, 25-44, 45-64, or ≥65 years), race/ethnicity (non-Hispanic white, non-Hispanic black or African American, Hispanic or Latino, or other), sexual orientation (heterosexual, homosexual, bisexual, or no response), annual household income ($20 000 increments up to ≥$100 000), educational level (less than high school, high school or General Education Diploma, some college or technical school, college graduate, or graduate or professional degree), marital status (single, widowed, separated or divorced, or married), and immigration status (foreign born: yes or no).

#### Potential Confounders

Past 12-month crime involvement was measured using 5 items, including injuring someone in a fight, committing robbery, carrying a weapon, stealing, and selling illegal drugs (Cronbach α = 0.87).^28^ Self-reported crime involvement measures are widely used in criminal justice research,^29,30^ including to address limitations and bias in official sources (eg, inaccurate reporting or lack of official recording of crimes).^31^ Crime involvement was recoded as a binary variable indicating any past 12-month involvement in criminal activities. Adverse childhood experiences were assessed using a 10-item Adverse Childhood Experience (ACE) questionnaire (Cronbach α = 0.83).^32^ A higher sum score indicates greater levels of adverse childhood experience exposure. Lifetime intimate partner violence was assessed (ie, physical, sexual, or psychological violence) using 15 dichotomous items from the National Intimate Partner and Sexual Violence Survey (NISVS),^33^ coded as a single dichotomous variable indicating any lifetime exposure.

### Statistical Analysis

All analyses were conducted using SPSS software, version 24 (IBM).^34^ Owing to forced choice responding, no missing cases were found for the main study variables of interest. Bivariate associations were assessed using χ^2^ tests or *t* tests. For the most salient demographics (gender identity, race/ethnicity, and sexual orientation),^6^ follow-up 2 × 2 χ^2^ tests were conducted and odds ratios (ORs) computed. Regression analyses were conducted to test adjusted associations between the independent variables (ie, each domain of the PPI) and the dependent variables (mental health outcomes). To create a parsimonious and consistent set of models, covariates were selected from among crime involvement, adverse childhood experiences, intimate partner violence, and sociodemographic variables by the significant association with each exposure and with at least 1 of the mental health outcomes. City was included in all models as a fixed effect. The ORs and 95% CIs for logistic regressions were produced by SPSS. An adjusted Cohen *d* was calculated for K6 for each exposure using the semipartial correlation from the regression models. All tests were 2-tailed and considered to be significant at an α of .05.

## Results

### Descriptive Statistics

Of the 1221 adults who consented to participate in the survey, 221 were immediately excluded owing to incorrectly responding to attention checks or discontinued participation. This yielded a final sample of 1000 respondents (81.9% participation rate). The demographic distribution fell within the planned sample quotas (±10% of US census demographics for each city) on race/ethnicity (non-Hispanic white, 339 [33.9%]; non-Hispanic black/African American, 390 [39.0%]; Hispanic/Latino, 178 [17.8%]; other, 93 [9.3%]), age (mean [SD], 39.8 [15.2] years), and gender (women, 600 [60.0%]; men, 394 [39.4%]; transgender, 6 [0.6%]), with a small overrepresentation of women in New York City and the 25- to 44-year age group in Baltimore and an underrepresentation of the oldest age group (≥65 years) in Baltimore (eTable 1 in the Supplement).

### Sociodemographic Distribution of Police Violence Exposures

**Figure 1.**
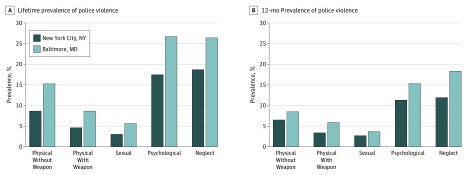
Lifetime Prevalence and 12-Month Prevalence of Police Violence Subtypes Among Adults in Baltimore, Maryland, and New York City, New York

The most common past 12-month and lifetime exposures were psychological (past 12 months, 132 respondents [13.2%]; lifetime, 216 respondents [21.6%]) and neglectful (past 12 months: 149 respondents [14.9%]; lifetime, 222 respondents [22.2%]), followed by the assaultive forms of violence: physical violence without a weapon (past 12 months, 75 respondents [7.5%]; lifetime, 117 respondents [11.7%]), physical violence with a weapon (past 12 months, 46 respondents [4.6%]; lifetime, 65 respondents [6.5%]), and sexual violence (past 12 months, 32 respondents [3.2%]; lifetime, 42 [4.2%]) (Figure 1).

Table 1 presents 12-month prevalence of police violence by sociodemographic group (for lifetime exposure, see eTable 2 in the Supplement). Differences were observed across the 3 gender identifications for 12-month physical (both with and without a weapon), sexual, and psychological violence. In 2 × 2 follow-ups (n = 994), men had more exposures than women for physical violence (OR, 3.50; 95% CI, 2.08-5.81; χ^2^ = 25; *P* < .001), physical violence with a weapon (OR, 2.53; 95% CI, 1.36-4.69; χ^2^ = 9.08; *P* = .003), and psychological violence (OR, 1.88; 95% CI, 1.3-2.72; χ^2^ = 11.3; *P* < .001); sexual violence was not significant (OR, 1.66; 95% CI, 0.79-3.47; χ^2^ = 1.82; *P* = .18). Despite the small number of transgender respondents (n = 6), 2 × 2 follow-up tests comparing transgender with nontransgender participants found large associations for 12-month sexual violence (OR, 33.28; 95% CI, 6.44-172; Fisher exact *P* = .001), physical violence (OR, 26; 95% CI, 4.68-144.4; Fisher exact *P* < .001), and physical violence with a weapon (OR, 10.8; 95% CI, 1.93-60.53; Fisher exact *P* = .028); psychological violence was not significant (OR, 3.32; 95% CI, 0.60-18.43; χ^2^ = 2.82; *P* = .14).

**Table 1.** Past 12-Month Prevalence of Police Violence by Confounding Factors, Including Bivariate Statistical Tests Among Adults in Baltimore, Maryland, and New York City, New York^a^

Variable	No. of Respondents	Physical Without a Weapon		Physical With a Weapon		Sexual		Psychological		Neglect	
		%	χ^2^	%	χ^2^	%	χ^2^	%	χ^2^	%	χ^2^
Gender											
Male	394	12.2	54.36^b^	6.9	20.11^b^	3.8	44.35^b^	17.5	13.34^b^	14.5	0.11
Female	600	3.8		2.8		2.3		10.2		15.2	
Transgender^c^	6	66.7		33.3		50.0		33.3		16.7	
Age group, y											
18-24	161	12.4	20.26^b^	7.5	10.35^b^	5.0	6.58	17.4	26.81^b^	16.1	14.14^b^
25-44	474	9.5		5.7		4.0		17.3		17.9	
45-64	288	2.8		2.1		1.4		6.9		12.5	
≥65	77	2.6		1.3		1.3		2.6		2.6	
Race/ethnicity											
White, non-Hispanic	339	2.7	17.91^b^	1.5	14.72^b^	1.2	7.37	5.9	28.27^b^	6.8	28.09^b^
Black/African American, non-Hispanic	390	9.5		7.4		4.6		19.2		20.0	
Hispanic/Latino	178	11.2		4.5		3.4		14.0		19.1	
Other	93	9.7		4.3		4.3		12.9		15.1	
Sexual orientation^d^											
Heterosexual	903	7.3	2.03	4.5	2.31	3.1	5.11	12.4	8.28^b^	15.0	0.041
Homosexual	28	14.3		10.7		10.7		28.6		14.3	
Bisexual	44	9.1		4.5		2.3		20.5		15.9	
Annual household income, $											
<20 000	209	11.5	10.62	7.7	8.02	3.8	1.95	13.4	14.76^b^	15.8	15.63^b^
20 000-39 999	211	7.1		4.6		3.3		18.0		19.4	
40 000-59 999	198	7.6		3.5		3.0		13.6		16.7	
60 000-79 999	138	8.7		5.8		4.3		15.9		17.4	
80 000-99 999	88	2.3		2.3		2.3		10.2		6.8	
≥100 000	156	4.5		2.6		1.9		5.1		7.7	
Education											
Less than high school	38	21.1	19.17^b^	13.2	14.45^b^	2.6	3.74	15.8	9.02	10.5	2.86
High school or GED	264	11.0		7.2		3.4		15.9		17.0	
Some college or technical school	287	4.9		2.8		2.4		15.7		16.0	
College graduate	279	5.7		3.9		4.7		10.4		12.9	
Graduate or professional degree	132	6.1		2.3		1.5		7.6		13.6	
Foreign-born											
Yes	120	5.0	1.23	3.3	0.50	0.8	2.47	8.3	2.82	10.8	1.78
No	880	7.8		4.8		3.5		13.9		15.5	
Marital status											
Single	505	9.5	8.61^b^	5.1	5.59	4.0	4.59	17.4	16.23^b^	17.6	6.26
Widowed	31	6.5		9.7		3.2		9.7		12.9	
Separated or divorced	110	1.8		0.9		0.0		7.3		13.6	
Married	354	6.5		4.5		3.1		9.3		11.6	
Crime involvement											
Yes	298	17.8	64.73^b^	12.8	64.28^b^	9.4	52.61^b^	29.9	102.91^b^	27.2	50.50^b^
No	702	3.1		1.1		0.6		6.1		9.7	
Adverse childhood experience											
Yes	564	9.8	9.46^b^	6.2	7.60^b^	4.8	10.52^b^	18.3	28.93^b^	20.9	37.00^b^
No	436	4.6		2.5		1.1		6.7		7.1	
Intimate partner violence											
Yes	481	11.6	22.92^b^	8.9	39.77^b^	6.2	27.60^b^	21.8	60.24^b^	21.8	35.10^b^
No	519	3.7		0.6		0.4		5.2		8.5	

Abbreviation: GED, General Education Diploma.

^a^ Degrees of freedom for gender were 2 (n = 1000); for age group, race/ethnicity, and marital status, 3 (n = 1000); for sexual orientation, 2 (n = 975); for annual household income, 5 (n = 1000); education, 4 (n = 1000); and for foreign born, crime involvement, adverse childhood experience, and intimate partner violence, 1 (n = 1000).

^b^ Statistically significant, *P* < .05 (2-tailed).

^c^ Prevalence rates among transgender respondents were based on a small subsample of respondents (n = 6) and should be interpreted with caution.

^d^ Respondents who opted not to respond to the sexual orientation item (n = 25) were not included in the χ^2^ analyses.

Rates of all types of violence were lowest for non-Hispanic white respondents; thus, follow-ups compared non-Hispanic white respondents with people of color. People of color were at greater risk than non-Hispanic white respondents, with ORs ranging from 4.4 (95% CI, 1.73-11.24; n = 100; χ^2^ = 11.41; *P* = .001) for physical violence with a weapon to 3.24 (95% CI, 2.03-5.16; n = 1000; χ^2^ = 26.6; *P* < .001) for police neglect, with physical violence without a weapon and psychological violence falling between (physical violence without a weapon: OR, 4.07; 95% CI, 2.0-8.26; χ^2^ = 17.35; *P* < .001; psychological violence: OR, 3.26; 95% CI, 1.98-5.34; χ^2^ = 23.86; *P* < .001).

Respondents who identified as homosexual or bisexual reported greater exposure to police violence, although only psychological violence was statistically significantly more common among sexual minorities in the tests across the 3 groups (heterosexual vs homosexual and bisexual: OR, 1.83; 95% CI, 1.08-3.12; χ^2^ = 5.16; *P* = .02). Because bisexual respondents reported a rate of sexual violence roughly equivalent to those who identified as heterosexual, a follow-up test found that homosexual participants were significantly more likely to report sexual violence than bisexual and heterosexual participants (OR, 3.9; 95% CI, 1.11-13.66; χ^2^ = 5.3; *P* = .02). Neither 2 × 2 follow-ups of physical violence or physical violence with a weapon comparing homosexual participants with all others were statistically significant.

All 12-month police violence exposures except sexual violence significantly varied by age and were most common among those 44 years or younger. Of the 1000 participants, 298 (29.8%) reported past 12-month crime involvement, nearly half (481 [48.1%]) of the sample reported lifetime experiences of intimate partner violence, and more than half (564 [56.4%]) reported 1 or more adverse childhood experience. Respondents reporting crime involvement, intimate partner violence, or adverse childhood experiences were all significantly more likely to be exposed to all subtypes of police violence.

### Association of Mental Health Outcomes With Police-Public Encounters

**Figure 2.**
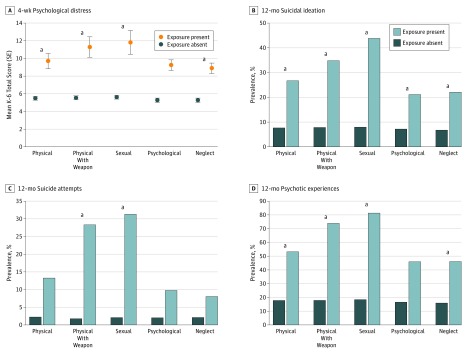
Four-Week Distress Scores and 12-Month Prevalence of Suicidal Ideation, Suicide Attempts, and Psychotic Experiences by Past 12-Month Police Violence Exposure Among Adults in Baltimore, Maryland, and New York City, New York All mental health outcomes were significantly associated with each police violence exposure in unadjusted χ^2^ analyses and *t* tests. A, Possible psychological distress scores ranged from 0 to 24, with higher scores indicating greater distress. ^a^Significant differences in adjusted analyses (α = .05).

Figure 2 shows the prevalence of each mental health outcome by each 12-month police violence exposure (for lifetime exposure, see eFigure 1 in the Supplement). The bivariate and adjusted Cohen *d*s for each exposure and K6 score are shown in Table 2. Cohen *d* expresses a mean difference in SD units, such that *d* = 1 would indicate that exposed respondents scored a mean 1 SD higher than nonexposed respondents. In the bivariate tests, the effect sizes of associations with psychological distress were large for all assaultive police exposures (physical violence without a weapon, physical violence with a weapon, and sexual violence) and medium for the others. All but psychological violence remained statistically significant in the adjusted analyses, although the effect sizes were small. Adjusted ORs for all exposure and outcome combinations (except K6 score) are presented in Table 3. The more assaultive forms of violence (ie, physical violence with a weapon and sexual violence) displayed the largest effect sizes for suicide attempts (physical violence with a weapon, OR, 7.30; 95% CI, 2.94-18.14; sexual violence, OR, 6.63; 95% CI, 2.64-16.64), suicidal ideation (physical violence with a weapon, OR, 2.72; 95% CI, 1.30-5.68; sexual violence, OR, 3.76; 95% CI, 1.72-8.20), and psychotic experiences (physical violence with a weapon, OR 4.34; 95% CI, 2.05-9.18; sexual violence, OR, 6.61; 95% CI, 2.52-17.36).

**Table 2.** Bivariate and Adjusted Cohen *d* for the Association of 12-Month Police Exposure With Psychological Distress Score Among Adults in Baltimore, Maryland, and New York City, New York^a^

Police Violence Exposure	Pooled Survey of Police-Public Encounters II Sample			
	Bivariate^a^		Adjusted^b^	
	Cohen *d*	*P* Value	Cohen *d*	*P* Value
Physical				
No weapon^c^	0.69	<.001	0.15	.009
With weapon^d^	0.91	<.001	0.16	.006
Sexual^e^	0.97	<.001	0.13	.02
Psychological^f^	0.64	<.001	0.08	.14
Neglectful^g^	0.58	<.001	0.12	.02

^a^ Psychological distress was measured by the Kessler Screening Scale for Psychological Distress (K6) score.

^a^ Bivariate Cohen *d* and *P* values from independent *t* tests.

^b^ Adjusted for Cohen *d* and *P* values from semipartial (part) correlations from multiple linear regression models.

^c^ Adjusted for gender, race/ethnicity, education, employment, crime, intimate partner violence, and city.

^d^ Adjusted for gender, employment, crime, intimate partner violence, and city.

^e^ Adjusted for crime, intimate partner violence, and city.

^f^ Adjusted for gender, race/ethnicity, foreign-born, crime, adverse childhood experiences, intimate partner violence, and city.

^g^ Adjusted for race/ethnicity, income, crime, adverse childhood experiences, intimate partner violence, and city.

**Table 3.** Fully Adjusted Logistic Regression Model of the Association Between 12-Month Police Exposure and Mental Health Outcomes Among Adults in Baltimore, Maryland, and New York City, New York

Outcome, by Police Violence Exposure	Pooled Survey of Police-Public Encounters II Sample	
	Odds Ratio (95% CI)	*P* Value
Physical		
No weapon^a^		
Suicidal ideation	2.45 (1.24-4.85)	.01
Suicide attempts	2.48 (0.97-6.38)	.06
Psychotic experiences	2.24 (1.26-3.99)	.006
With weapon^b^		
Suicidal ideation	2.72 (1.30-5.68)	.008
Suicide attempts	7.30 (2.94-18.14)	<.001
Psychotic experiences	4.34 (2.05-9.18)	<.001
Sexual^c^		
Suicidal ideation	3.76 (1.72-8.20)	.001
Suicide attempts	6.63 (2.64-16.64)	<.001
Psychotic experiences	6.61 (2.52-17.36)	<.001
Psychological^d^		
Suicidal ideation	1.73 (1.01-2.96)	.047
Suicide attempts	1.65 (0.76-3.59)	.21
Psychotic experiences	1.54 (0.98-2.40)	.06
Neglect^e^		
Suicidal ideation	2.15 (1.27-3.62)	.004
Suicide attempts	1.63 (0.71-3.71)	.25
Psychotic experiences	2.45 (1.41-3.74)	<.001

^a^ Adjusted for gender, race/ethnicity, education, employment, crime, intimate partner violence, and city.

^b^ Adjusted for gender, employment, crime, intimate partner violence, and city.

^c^ Adjusted for crime, intimate partner violence, and city.

^d^ Adjusted for gender, race/ethnicity, crime, adverse childhood experiences, intimate partner violence, and city.

^e^ Adjusted for race/ethnicity, income, crime, adverse childhood experiences, intimate partner violence, and city.

## Discussion

Police violence in the United States constitutes a public health problem that is important to address because of the expanse of its potential mental health consequences and the limited attention it has received from policy makers and researchers. Our main findings were that 12-month police violence exposures were commonly reported among adult residents of Baltimore and New York City; communities of color and lesbian, gay, bisexual, and transgender communities were disproportionately affected, and these exposures were associated with greater odds of current psychological distress and concurrent (12-month) suicidal ideation, suicide attempts, and psychotic experiences. When adjusted for other forms of violence exposure, suicidal ideation remained significantly associated with all forms of police violence, whereas suicide attempts and psychotic experiences were primarily associated with severe assaultive forms of police violence (ie, physical violence with a weapon, sexual violence), with psychotic experiences additionally associated with neglectful policing. Psychological distress was associated with all forms of police violence exposure except psychological in the adjusted analyses.

Findings from SPPE II further document the prevalence of police violence exposure in the general population^6,7,8,11,12^ and provide needed data on recent (past 12-month) exposure to police violence, including its disproportionate effect on males and racial/ethnic minority subpopulations. Previously in the SPPE I, lifetime police violence exposure was shown to be widespread in 4 US cities and disproportionately experienced by respondents who are African American or Latino, male or transgender, younger, or less educated and by those with lower incomes.^6^ This pattern of findings was replicated in the SPPE II data using past 12-month exposures, with minor divergences in prevalence but not in the overall pattern of demographic variation. Police violence was disproportionately directed toward disadvantaged groups (eg, 9.5% of black respondents reported exposure to physical police violence compared with 2.7% of white respondents). Furthermore, although there was a small sample of transgender participants, the ORs for police violence were high, suggesting an increased vulnerability of this population.

Of note, findings from SPPE II suggest that recent sexual police violence and physical police violence with a weapon may be among the strongest correlates of adverse mental health outcomes. The odds for recent suicide attempts and psychotic experiences were nearly 7-fold greater among those exposed to sexual violence by police in the past 12 months (suicide attempts, OR, 6.63; 95% CI, 2.64-16.64; psychotic experiences, OR, 6.61; 95% CI, 2.52-17.36). Among those exposed to physical violence with a weapon in the past 12 months, the odds for recent suicide attempts were more than 7-fold greater (OR, 7.30; 95% CI, 2.94-18.14). The null or modest adjusted associations with psychological violence (which was significantly associated with distress and psychosis in SPPE I)^6,7^ are more difficult to interpret, because this exposure may be biased across demographic groups.

This study provides empirical results suggesting elevated odds for psychological distress, suicidal behavior, and psychotic experiences among individuals recently exposed to police violence. Findings suggest the need for clinicians to be attuned to the prevalence of police violence among patient populations, especially racial/ethnic minorities, males, young adults, and economically disadvantaged individuals. Crisis Intervention Team approaches, which have been implemented in New York City and Baltimore,^35,36^ may be effective in reducing incidents of police violence when intervening with people with mental illness,^37,38^ although it is not clear from our data what proportion of individuals endorsing violence exposure in this study had been diagnosed with a mental illness or whether these incidents arose from crisis situations. This information may be useful for policy makers in determining interventions to reduce the rate of police violence exposures, particularly in underserved communities. Future research is needed to understand causal pathways between police violence and mental health symptoms to better inform clinical responses to individuals with experiences of police violence (eAppendix and eFigure 2 in the Supplement).

### Limitations

There are several potential limitations of this study. First, causal pathways cannot be established owing to the study’s cross-sectional design, and longitudinal research is needed to assess police violence exposures over time. Second, challenges in achieving sampling quotas were encountered, resulting in oversampling of some subgroups (ie, women in New York City and younger respondents in Baltimore), and quota sampling does not yield truly representative samples as in probability sampling. Given that online samples are less likely to capture some vulnerable subpopulations who may be at greater risk of police violence exposure (eg, homeless or institutionalized individuals or those with very low income), it is likely that the police violence exposure rates reported in this study are a lower bound or “floor” to the actual community-level prevalence. Still, these findings were largely consistent with SPPE I results and other studies on police violence using general population samples.^6,7,8,11,12^

Both SPPE I and SPPE II data found elevated prevalence rates of police violence exposure among their very small subsamples of transgender individuals, which should be replicated in larger samples owing to the putative high risk of exposure among this group. Disparities in exposure by sexual orientation should be further explored in future studies that are amply powered to address this question. In addition, even though there were adjustments for substance use in the context of illegal activities, adjustments were not made for related potential confounders, including alcohol use or clinician-assessed psychiatric disorder. It will also be important to expand surveys of police violence into other languages in the future, the exclusion of which may have biased the subsample from New York City (where many residents exclusively speak Spanish or other foreign languages). Although previously validated measures of mental health outcomes were used, there is extensive comorbidity across mental health symptoms and we may not be able to consider these outcomes to be independent of one another.

Also, the situational context of police violence exposures or circumstances contributing to the police encounter or arrest was not clear. For example, it is unclear whether participants experienced physical violence from police owing to involvement in crimes or while resisting arrest, although there were adjustments for past 12-month crime involvement to control for this possibility. Furthermore, situational context is likely relevant only for physical police violence because psychological violence (eg, racial slurs), neglect (eg, failure to respond when called), and especially sexual violence by police are unjustifiable under any circumstances. Future studies can further clarify this by assessing the total number of interactions with police for each individual to determine whether people with psychiatric symptoms are at greater risk for interacting with police in general or at greater risk for each interaction becoming violent.

## Conclusions

Police violence appeared to be commonly reported, especially among racial/ethnic and sexual minorities. Associations between violence and mental health outcomes did not appear to be explained by confounding factors and seemed to be especially pronounced for assaultive forms of violence.

## Notes

Supplement. **eTable 1.** Sociodemographic Descriptives of the Sample, by City **eTable 2.** Lifetime Prevalence of Police Violence by Sociodemographic Group, Including Bivariate Statistical Tests **eFigure 1.** Mean 4-Week Distress Scores (A) and 12-Month Prevalence of (B) Suicidal Ideation, (C) Suicide Attempts, and (D) Psychotic Experiences by Lifetime Police Violence Exposure **eAppendix.** Brief Discussion of Potential Causal Interpretations of Study Findings **eFigure 2.** Hypothetical Data Illustrating Predicted Patterns of Associations Between Each Exposure and Mental Health Based on (A) Police Violence Causing Psychiatric Symptoms, (B) Psychiatric Symptoms Causing Increased Police Contact, and (C) a Lack of Causal Associations **eReferences.**
