# Supplementary material for: Association of Exposure to Police Violence With Prevalence of Mental Health Symptoms Among Urban Residents in the United States
Source: JAMA Netw Open. 2018 Nov 21;1(7):e184945. doi: 10.1001/jamanetworkopen.2018.4945 (PMC6324385; doi:10.1001/jamanetworkopen.2018.4945)
Supplement: Supplement. — eTable 1. Sociodemographic Descriptives of the Sample, by City eTable 2. Lifetime Prevalence of Police Violence by Sociodemographic Group, Including Bivariate Statistical Tests eFigure 1. Mean 4-Week Distress Scores (A) and 12-Month Prevalence of (B) Suicidal Ideation, (C) Suicide Attempts, and (D) Psychotic Experiences by Lifetime Police Violence Exposure eAppendix. Brief Discussion of Potential Causal Interpretations of Study Findings eFigure 2. Hypothetical Data Illustrating Predicted Patterns of Associations Between Each Exposure and Mental Health Based on (A) Police Violence Causing Psychiatric Symptoms, (B) Psychiatric Symptoms Causing Increased Police Contact, and (C) a Lack of Causal Associations eReferences. [file jamanetwopen-1-e184945-s001.pdf]

Supplementary Online Content

DeVylder JE, Jun H-J, Fedina L, et al. Association of exposure to police violence with prevalence of mental health symptoms among urban residents in the United States. *JAMA Netw Open*. 2018;1(7):e184945. doi:10.1001/jamanetworkopen.2018.4945

eTable 1. Sociodemographic Descriptives of the Sample, by City
eTable 2. Lifetime Prevalence of Police Violence by Sociodemographic Group, Including Bivariate Statistical Tests
eFigure 1. Mean 4-Week Distress Scores (A) and 12-Month Prevalence of (B) Suicidal Ideation, (C) Suicide Attempts, and (D) Psychotic Experiences by Lifetime Police Violence Exposure
eAppendix. Brief Discussion of Potential Causal Interpretations of Study Findings
eFigure 2. Hypothetical Data Illustrating Predicted Patterns of Associations Between Each Exposure and Mental Health Based on (A) Police Violence Causing Psychiatric Symptoms, (B) Psychiatric Symptoms Causing Increased Police Contact, and (C) a Lack of Causal Associations
eReferences.

This supplementary material has been provided by the authors to give readers additional information about their work.

eTable 1. Sociodemographic Descriptives of the Sample, by City. Values indicate n (%)

	New York City	Baltimore	Total Sample
	n = 550	n = 450	N = 1,000
**Gender**			
Male	211 (38.4)	183 (40.7)	394 (39.4)
Female	337 (61.3)	263 (58.4)	600 (60.0)
Trans	2 (0.4)	4 (0.9)	6 (0.6)
**Age group**			
18-24	97 (17.6)	64 (14.2)	161 (16.1)
25-44	223 (40.5)	251 (55.8)	474 (47.4)
45-64	174 (31.6)	114 (25.3)	288 (28.8)
65+	56 (10.2)	21 (4.7)	77 (7.7)
**Race**			
White, non-Latino	204 (37.1)	135 (30.0)	339 (33.9)
Black, non-Latino	144 (26.2)	246 (54.7)	390 (39.0)
Latino	143 (26.0)	35 (7.8)	178 (17.8)
Other	59 (10.7)	34 (7.6)	93 (9.3)
**Sexual orientation**			
Heterosexual	498 (90.5)	405 (90.0)	903 (90.3)
Homosexual	17 (3.1)	11 (2.4)	28 (2.8)
Bisexual	20 (3.6)	24 (5.3)	44 (4.4)
Not specified	15 (2.7)	10 (2.2)	25 (2.5)
**Annual household income**			
< 20,000	97 (17.6)	112 (24.9)	209 (20.9)
20,000-39,999	102 (18.5)	109 (24.2)	211 (21.1)
40,000-59,999	100 (18.2)	98 (21.8)	198 (19.8)
60,000-79,999	90 (16.4)	48 (10.7)	138 (13.8)
80,000-99,999	58 (10.5)	30 (6.7)	88 (8.8)
≥ 100,000	103 (18.7)	53 (11.8)	158 (15.8)
**Education**			
< High school	18 (3.3)	20 (4.4)	38 (3.8)
High school / GED	121 (22.0)	143 (31.8)	264 (26.4)
Some college / tech	152 (27.6)	135 (30.0)	287 (28.7)
College graduate	180 (32.7)	99 (22.0)	279 (27.9)
Grad/professional	79 (14.4)	53 (11.8)	132 (13.2)
**Foreign-born**			
Yes	83 (15.1)	37 (8.2)	120 (12.0)
No	467 (84.9)	413 (91.8)	880 (88.0)
**Marital status**			
Single	260 (47.3)	245 (54.4)	505 (50.5)
Widowed	21 (3.8)	10 (2.2)	31 (3.1)
Separated/divorced	54 (9.8)	56 (12.4)	110 (11.0)
Married	215 (39.1)	139 (30.9)	354 (35.4)

© DeVylder JE et al. *JAMA Network Open*.

eTable 2. Lifetime Prevalence of Police Violence by Sociodemographic Group, Including Bivariate Statistical Tests

	Physical	Physical w/ weapon	Sexual	Psychological	Neglect
**Gender**	$X^2_{2,1000)}=57.30$ *p* < .001	$X^2_{(2,1000)}=24.93$ *p* < .001	$X^2_{(2,1000)}=33.62$ *p* < .001	$X^2_{(2,1000)}=10.60$ *p* = .005	$X^2_{(2,1000)}=1.17$ *p* = .56
Male	19.3	10.4	5.1	26.6	20.6
Female	6.2	3.7	3.2	18.2	23.3
Trans	66.7	33.3	50.0	33.3	16.7
**Age group**	$X^2_{3,1000)}=20.63$ *p* < .001	$X^2_{(3,1000)}=18.57$ *p* < .001	$X^2_{(3,1000)}=9.37$ *p* = .025	$X^2_{(3,1000)}=29.52$ *p* < .001	$X^2_{(3,1000)}=14.55$ *p* = .002
18-24	14.9	10.6	5.6	21.1	24.8
25-44	15.2	8.4	5.7	28.3	25.5
45-64	6.6	2.4	1.7	14.6	19.1
65+	2.6	1.3	1.3	7.8	7.8
**Race**	$X^2_{(3,1000)}=33.68$ *p* < .001	$X^2_{(3,1000)}=23.67$ *p* < .001	$X^2_{(3,1000)}=6.27$ *p* = .099	$X^2_{(3,1000)}=42.38$ *p* < .001	$X^2_{(3,1000)}=34.55$ *p* < .001
White, non-Hispanic	4.1	2.4	2.4	10.9	11.8
Black, non-Hispanic	17.7	11.0	5.9	30.8	29.0
Hispanic/Latino	14.0	5.6	3.4	22.5	27.5
Other	9.7	4.3	5.4	20.4	21.5
**Sexual orientation**	$X^2_{(2,1000)}=3.31$ *p* = .19	$X^2_{(2,1000)}=3.04$ *p* = .22	$X^2_{(2,1000)}=2.89$ *p* = .24	$X^2_{(2,1000)}=9.70$ *p* = .008	$X^2_{(2,1000)}=3.74$ *p* = .15
Heterosexual	11.4	6.4	4.1	20.6	21.8
Homosexual	21.4	14.3	10.7	39.3	25.0
Bisexual	15.9	4.5	4.5	34.1	34.1
**Annual household income**	$X^2_{(5,1000)}=12.70$ *p* < .001	$X^2_{(5,1000)}=8.55$ *p* = .003	$X^2_{(5,1000)}=1.11$ *p* = .29	$X^2_{(5,1000)}=9.09$ *p* = .003	$X^2_{(5,1000)}=8.89$ *p* = .003
< 20,000	18.7	10.5	5.3	24.4	24.9
20,000-39,999	11.4	6.6	4.3	24.2	26.1
40,000-59,999	11.6	6.1	4.0	24.2	22.7
60,000-79,999	10.9	7.2	5.1	22.5	25.4
80,000-99,999	5.7	2.3	2.3	19.3	15.9
> 100,000	7.1	3.2	3.2	11.5	13.5
**Education**	$X^2_{(4,1000)}=25.43$ *p* < .001	$X^2_{(4,1000)}=15.33$ *p* < .001	$X^2_{(4,1000)}=.642$ *p* = .42	$X^2_{(4,1000)}=9.17$ *p* = .002	$X^2_{(4,1000)}=2.60$ *p* = .11
< High school	26.3	15.8	2.6	28.9	21.1
High school / GED	18.9	11.0	4.9	25.0	25.4
Some college / tech	9.8	4.9	4.2	24.4	23.3
College graduate	7.2	3.9	5.0	17.6	19.7
Grad/professional	6.8	3.8	1.5	15.2	18.9
**Foreign-born**	$X^2_{(1,1000)}=2.33$ *p* = .13	$X^2_{(1,1000)}=2.25$ *p* = .13	$X^2_{(1,1000)}=3.84$ *p* = .050	$X^2_{(1,1000)}=4.45$ *p* = .035	$X^2_{(1,1000)}=1.74$ *p* = .19
Yes	7.5	3.3	0.8	14.2	17.5
No	12.3	6.9	4.7	22.6	22.8

© DeVylder JE et al. *JAMA Network Open*.

**Marital status**	$X^2_{(3,1000)}$=8.27 *p* = .041	$X^2_{(3,1000)}$=3.99 *p* = .26	$X^2_{(3,1000)}$=6.03 *p* = .11	$X^2_{(3,1000)}$=9.43 *p* = .024	$X^2_{(3,1000)}$=3.58 *p* = .31
Single	14.5	7.7	5.1	25.5	24.4
Widowed	12.9	9.7	3.2	16.1	22.6
Separated/divorced	7.3	3.6	0.0	17.3	22.7
Married	9.0	5.4	4.2	17.8	18.9
**Crime involvement**	$X^2_{(1,1000)}$=70.79 *p* < .001	$X^2_{(1,1000)}$=69.98 *p* < .001	$X^2_{(1,1000)}$=45.06 *p* < .001	$X^2_{(1,1000)}$=87.28 *p* < .001	$X^2_{(1,1000)}$=43.89 *p* < .001
Yes	24.8	16.4	10.7	40.3	35.6
No	6.1	2.3	1.4	13.7	16.5
**Adverse childhood experience**	$X^2_{(1,1000)}$=12.76 *p* < .001	$X^2_{(1,1000)}$=13.75 *p* < .001	$X^2_{(1,1000)}$=15.31 *p* < .001	$X^2_{(1,1000)}$=53.39 *p* < .001	$X^2_{(1,1000)}$=68.06 *p* < .001
Yes	14.9	9.0	6.4	30.0	31.7
No	7.6	3.2	1.4	10.8	9.9
**Intimate partner violence**	$X^2_{(1,1000)}$=31.81 *p* < .001	$X^2_{(1,1000)}$=35.25 *p* < .001	$X^2_{(1,1000)}$=37.47 *p* < .001	$X^2_{(1,1000)}$=77.13 *p* < .001	$X^2_{(1,1000)}$=0.72 *p* < .001
Yes	17.6	11.2	8.2	33.3	32.7
No	6.1	2.0	0.4	10.4	12.2

eFigure 1. Mean 4-week distress scores (A) and 12-month prevalence of (B) Suicidal ideation, (C) Suicide Attempts, and (D) Psychotic Experiences by lifetime Police Violence Exposure

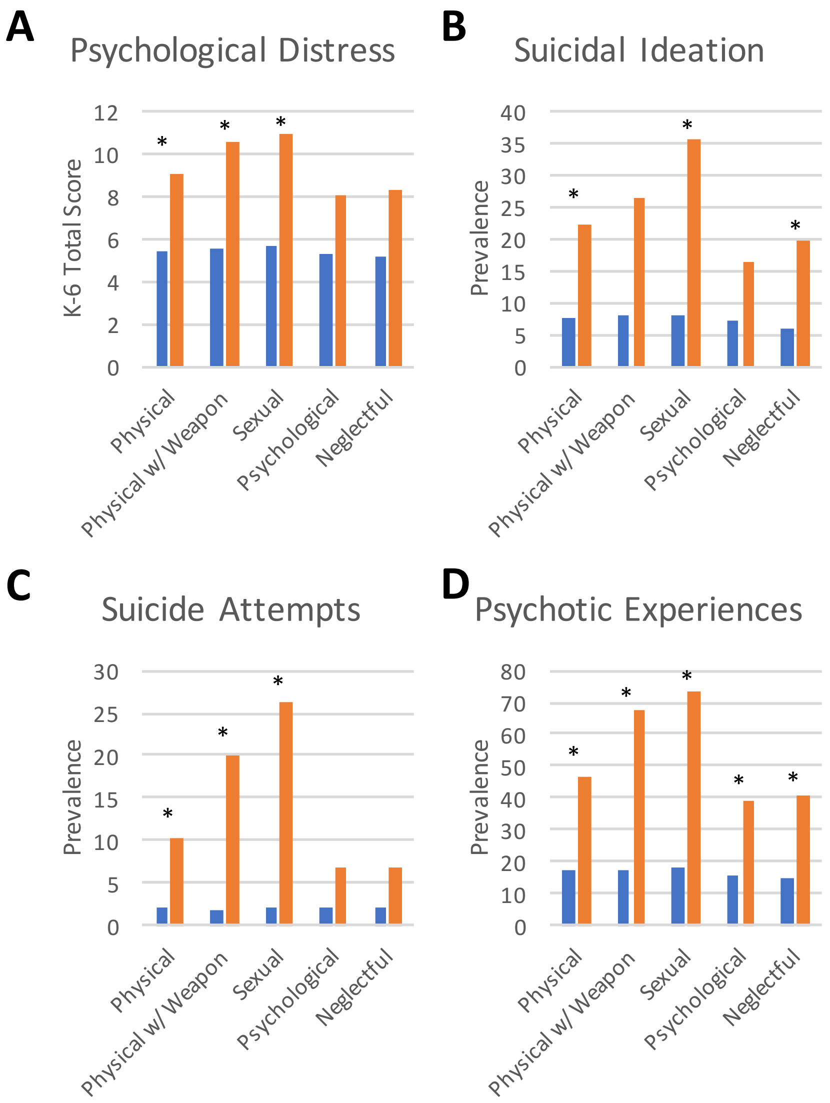

Orange bars indicate the presence of each violence exposure, whereas blue bars indicate its absence. Possible psychological distress scores (A) ranged from 0-24, with higher scores indicating greater distress. Asterisks indicate significant differences in adjusted analyses. All mental health outcomes were significantly associated with each lifetime police violence exposure in unadjusted chi-square analyses and t-tests.

© DeVylder JE et al. *JAMA Network Open*.

eAppendix. Brief discussion of potential causal interpretations of study findings

The use of cross-sectional data limits causal inference regarding whether police violence is acting as a psychogenic stressor, whether mental health symptoms are drawing police attention and subsequent violence, or whether these associations are driven by related underlying factors. However, each of these explanations can be expected to yield unique patterns of associations, even in cross-sectional data. To the extent that mental health symptoms are leading to an elevated risk for police violence exposure, the most likely causal pathway would be one in which symptoms draw attention and lead to greater police contact, and greater frequency of contacts would lead to greater levels of violence exposure, even if the rate of exposure per contact was unchanged. Such mechanisms have been used to explain racial disparities in police violence, for which it has been proposed that police respond to individuals similarly regardless of race, but disproportionately interact with people of color compared to Whites.[s1,s2] However, this causal pathway would likely lead to a uniformly greater violence exposure across all sub-types. Alternatively, if police violence is leading to psychological distress and mental health symptoms, we would expect the strongest relationship between those outcomes and the most traumatic forms of exposure (i.e., physical violence with a weapon and sexual violence), and weaker association with less invasive forms of violence. This is what we found. While this does not substitute for the value of prospective longitudinal data, results from these cross-sectional data do appear more consistent with police violence serving as a risk exposure for severe mental health outcomes (see **eFigure 2**).

eFigure 2. Hypothetical Data Illustrating Predicted Patterns of Associations Between Each Exposure and Mental Health Based on (A) Police Violence Causing Psychiatric Symptoms, (B) Psychiatric Symptoms Causing Increased Police Contact, and (C) A Lack of Causal Associations[a]

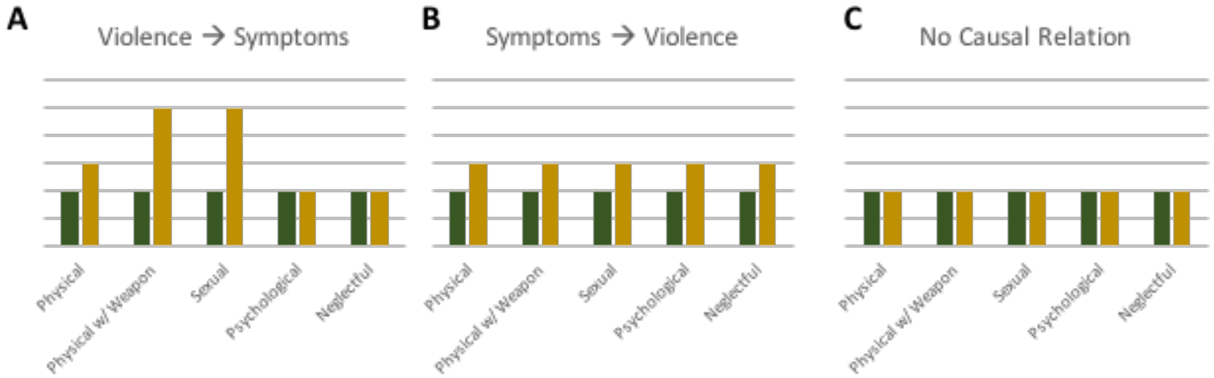

[a] Findings from the present study were most consistent with pattern A for suicide attempts and psychotic experiences, but were less clear for suicidal ideation and psychological distress.

© DeVylder JE et al. *JAMA Network Open*.

eReferences.

1. Fryer Jr RG. *An empirical analysis of racial differences in police use of force*. National Bureau of Economic Research; 2016.

2. Miller TR, Lawrence BA, Carlson NN, et al. Perils of police action: a cautionary tale from US data sets. *Injury prevention.* 2017;23(1):27–32.
